# Long-term effects of neoadjuvant chemotherapy in variant histology locally advanced colon cancer: a propensity score-matched analysis

**DOI:** 10.1080/15384047.2024.2441511

**Published:** 2024-12-24

**Authors:** Qiancheng Wang, Shiyang Jin, Zeshen Wang, Yuming Ju, Kuan Wang

**Affiliations:** Department of Gastrointestinal Surgery, Harbin Medical University Cancer Hospital, Harbin, China

**Keywords:** Colectomy, colon cancer, neoadjuvant chemotherapy, survival

## Abstract

**Purpose:**

Neoadjuvant chemotherapy (NAC) has proven valuable in treating locally advanced colon cancer (LACC) and is included as a treatment option for patients with clinical T4b colon cancer by the National Comprehensive Cancer Network. However, the long-term survival benefit of NAC in LACC remains debated, due to a lack of conclusive clinical trial results identifying the patients who would benefit most from NAC. This study aimed to assess the efficacy of NAC in patients with LACC based on histological subtype.

**Patients and methods:**

This retrospective study analyzed 3,709 patients with LACC who underwent curative resection at Harbin Medical University Cancer Hospital between 2014 and 2018. Patients were grouped into two groups: neoadjuvant chemotherapy (NAC) and adjuvant chemotherapy (AC) groups. Propensity score matching (PSM) was used to adjust for confounders, and survival outcomes of the two groups across different histological subtypes were evaluated using Kaplan-Meier (K-M) curves and log-rank tests.

**Results:**

Patients with non-mucinous adenocarcinoma (NMAC) treated with NAC had a significantly improved 5-year OS rate (76.3% vs. 69.2%, *p* = .039) and DFS rate (67.2% vs. 60.1%, *p* = .041) compared with patients treated with AC. However, there was no significant difference in OS and DFS between the two treatment groups among patients with mucinous adenocarcinoma (MAC) and signet ring cell carcinoma (SRCC).

**Conclusion:**

In patients with LACC, the prognostic value of NAC varied by histology. NMAC may serve as a predictor of improved long-term survival benefit from NAC in these patients.

## Introduction

Colon cancer (CC) is the third most common cancer and the second leading cause of cancer related death worldwide.^1^ About one quarter of these patients present with locally advanced colon cancer (LACC) without signs of distant metastases.^1,2^ LACC is defined as high-risk T3 (≥5 mm invasion beyond the muscularis propria) and T4 tumors, with or without nodal involvement. Currently, complete tumor resection followed by adjuvant chemotherapy (AC) remains the standard treatment for LACC.^3^ However, despite this aggressive therapeutic strategy with curative intent, the prognosis of patients remains unsatisfactory, recurrence varies from 20 to 30%, reflecting the relative failure of such a strategy in eradicating micrometastases of tumor cells.^4^ Therefore, there is an urgent need to explore an alternative treatment for patients with LACC.

In recent years, NAC has gradually become an accepted standard therapy for solid tumors such as mammary, esophageal, gastric, and rectal cancer.^5–8^ Compared to AC, NAC, administered before surgery, can effectively promote tumor regression, reduce tumor size, and improve the resection margin, thus increasing the likelihood of achieving an R0 resection.^9^ Another potential benefit of preoperative NAC is its ability to reduce the dissemination of tumor cells during surgery and eliminate small, occult metastases through early intervention, which may reduce the risk of recurrence.^10^ Furthermore, some patients may be unable to complete adjuvant chemotherapy as scheduled due to severe postoperative complications. In such cases, neoadjuvant chemotherapy allows for the assessment of the tumor’s response to treatment prior to surgery, thereby providing more precise guidance for subsequent therapeutic decisions. As a result, the proportion of patients who receive and successfully complete NAC is typically higher than that of patients who undergo postoperative adjuvant chemotherapy.

Although well established and widely applied in the majority of gastrointestinal tumors, the use of NAC in the field of non-metastatic LACC remains controversial. Several recent researches have reported the feasibility and efficacy of NAC in LACC.^11–14^ These studies demonstrated significantly histological tumor regression with acceptable toxicity and low perioperative morbidity when LACC patients are treated with NAC. However, most studies have focused on short-term postoperative outcomes and the quality of surgical resections in LACC patients. Until now, little has been known about the impact of NAC on the long-term prognosis in LACC patients. In a retrospective analysis of 2146 patients with CC classified as T4, Gooyer et al. reported no significant difference in 5-year OS between the NAC and control groups.^9^ Moreover, several publicly available randomized controlled trials, including the FOXTROT and PRODIGE22 trials, also failed to demonstrate the long-term oncological benefits of NAC for LACC patients.^15,16^ Relevant research has shown that patients with different phenotypes and characteristics may have had different sensitivities to chemotherapy drugs.^9,17,18^ One of the strategies for improving the efficacy of NAC is to predict drug sensitivity. The histological classification of tumors is an important indicator for evaluating tumor occurrence, development, and prognosis. Different histological types have different growth rates and invasiveness, and play a pivotal role in formulating individualized treatment plans for various tumors. For example, chemotherapy regimens vary for each histological type of urinary cancer. Carboplatin/cyclophosphamide is recommended for carcinosarcoma, paclitaxel/cisplatin for clear cell carcinoma, and paclitaxel/carboplatin for malignant stromal tumors.^19–21^ Given the biological and clinicopathologic differences between patients with different histological classifications, it may be valuable to further investigate whether histological classification affects the role of NAC in LACC.

Therefore, we conducted this population-based study aimed to investigate the impact of NAC on long-term survival in patients with LACC based on histological types.

## Patients and methods

### Cohort selection

**Figure 1.**
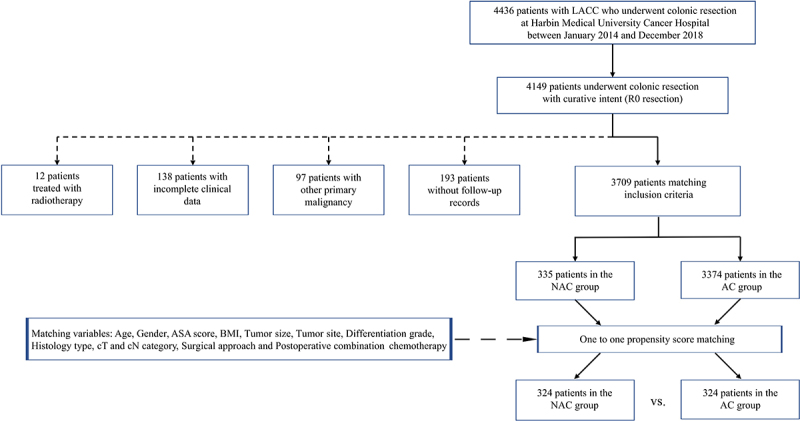
Flowchart of patient selection.

The study retrospectively collected clinical data from 4436 patients with locally advanced colon cancer (LACC) who underwent colectomy at Harbin Medical University Cancer Hospital between January 2014 and December 2018 (Figure 1). The staging criteria were based on the eighth edition of the American Cancer Society’s TNM staging system for colorectal cancer. This study was approved by the Institutional Review Committee of Harbin Medical University Cancer Hospital (Ethics number: 2023–150), and written informed consent for participation was obtained from all participants.

Patients needed to satisfy the following inclusion and exclusion criteria. The inclusion criteria were: age ≥18 years, pathologically confirmed LACC, no evidence of distant metastasis, curative resection (R0), and complete clinicopathological data along with cancer-specific survival records. The exclusion criteria were: patients who received radiotherapy, those with significant perioperative complications, and those with a history of other primary malignancies. Patients were then stratified into two groups based on the timing of chemotherapy relative to surgery: the NAC group (chemotherapy followed by surgery) and the AC group (surgery followed by chemotherapy).

A total of 3709 patients with LACC met the inclusion criteria and were included in the retrospectively analysis: the NAC group (*n* = 335) and the AC group (*n* = 3374) (Figure 1). The remaining 727 patients were excluded based on the following criteria: 287 patients with non-R0 resection, 193 patients without follow-up records, 97 patients with other primary malignancies, 138 patients with incomplete clinical data, and 12 patients who had received radiotherapy.

### Study variables

The following variables were included in the extracted data: age, gender, American Society of Anesthesiologists (ASA) score, body mass index (BMI), tumor site, tumor size, differentiation grade, morphology, clinical T category (cT), clinical N category (cN), surgical approach and postoperative chemotherapy regimen.

In the present study, age was regrouped into ≤65 and >65; gender was classified as male and female; ASA score was regrouped into <3 and ≥3. The tumor site was grouped into left colon (colonic splenic flexure, descending colon, and sigmoid colon) and right colon (ascending colon and colonic hepatic flexure); the tumor size was divided into <5 cm and ≥5 cm; the differentiation grade was classified as “well/moderate” and “poor/undifferentiated”; the morphology variable was classified as “non-mucinous adenocarcinoma (NMAC)”, “mucinous adenocarcinoma (MAC)” and “signet ring cell carcinoma (SRCC)”; Surgical approach was recorded as “open” or “laparoscopic”. Postoperative chemotherapy was classified as “single-agent chemotherapy” or “combination chemotherapy”.

### Chemotherapeutic regimen

In this study, patients in the NAC group received at least four cycles of the XELOX chemotherapy regimen prior to surgery, while those in the AC group received either the XELOX regimen or capecitabine monotherapy following surgery. The XELOX regimen consists of capecitabine and oxaliplatin. Each cycle included 14 days of oral capecitabine administration, with intravenous oxaliplatin administered on Day 1 of each cycle. Oxaliplatin was given at a dose of 130 mg/m^2^ on Day 1 of each cycle, and capecitabine was administered at a dose of 1250 mg/m^2^, divided into two doses and taken twice daily for 14 days. The capecitabine monotherapy regimen involved oral administration twice daily at a dose of 1250 mg/m^2^ for 5 consecutive days, followed by a 2-day treatment break, constituting one treatment cycle. The total duration of treatment was 6 months. All participants in this study successfully completed the prescribed chemotherapy regimens.

### Outcomes and follow‐up

The primary outcome of this study was overall survival (OS), defined as the time from diagnosis to death from any cause. The secondary outcome was disease-free survival (DFS), which was defined as the time from diagnosis to the first recurrence (local, regional, or metastatic), the occurrence of a second cancer, or death.

All patients were followed up with tumor markers (CEA, CA19–9, CA125, and CA242) and clinical examinations every 3 months during the first 3 years, and every 6 months for the following 2 years. Abdominal-pelvic CT scans were performed every 6 months, and colonoscopy was conducted annually. The final follow-up took place in September 2023.

### Statistical analysis

Continuous variables were reported as median and interquartile range (IQR), while categorical variables were described with frequencies and percentages. The Comparison of baseline clinicopathological variables between subgroups was performed using the two independent sample t-test, χ^2^ test, or Fisher’s exact test. Followed by propensity score matching (PSM) to minimize the potential confounding factors due to nonrandomized assignment within two treatment groups. The PSM analysis included all baseline characteristics that were significantly associated to both the choice of either NAC group or AC group and all unbalanced baseline covariables. Variables used in PSM were: age, gender, ASA score, BMI, tumor site, tumor size, differentiation grade, morphology, clinical T category, clinical N category, surgical approach and postoperative combination chemotherapy. Patients were matched in a 1:1 ratio with the ‘nearest match’ method and a maximum caliber of 0.01. After PSM, baseline characteristics were compared to assure that no significantly differences persisted between the subgroups. The standardized mean differences (SMD) before and after PSM were calculated to measure balance between groups. A SMD of >0.1 was considered as an indicator for remaining confounding. Survival curves were plotted using the Kaplan-Meier (K-M) method and compared with the log-rank test for each histological types (NMAC, MAC and SRCC). Additionally, A landmark analysis was conducted to account for immortal time bias, with six months post-diagnosis defined as the landmark time point. Patients lost to follow-up or who died within six months of diagnosis were excluded from further analysis in the landmark cohort. All p-values were two-tailed and considered to indicate statistical significance if *p* < .05. Statistical analysis was performed using Statistical Package for Social Sciences (SPSS) software, version 27 and R software (version 4.2.3; R Foundation for Statistical Computing, Vienna, Austria).

## Results

### Patients

A total of 3709 patients with LACC met inclusion criteria and were included in the present study: NAC group (*n* = 335) and AC group (*n* = 3374) (Figure 1). The main causes of NAC included reducing the tumor burden to improve the complete resection rate (cT4b: 80/335, 23.88%) and multiple local lymph node metastases (168/335, 50.15%). The characteristics of the entire cohort are presented in Table 1. There were significant differences in patient demographics, tumor features, and treatment between the two groups.

**Table 1.** Baseline and tumor characteristics of NAC group, compared to the AC group, raw and matched data.

	Raw data			PSM adjusted data			
	NAC (*n* = 335)	AC (*n* = 3374)	*p* value	NAC (*n* = 324)	AC (*n* = 324)	*p* value	SMD
Age, n (%)			<0.001			0.207	0.035
≤65	238 (71.04)	1875 (55.57)		227 (70.06)	212 (65.43)		
>65	97 (28.96)	1499 (44.43)		97 (29.94)	112 (34.57)		
Gender, n (%)			0.901			0.983	0.009
Male	205 (61.19)	2053 (60.85)		200 (61.73)	193 (59.57)		
Female	130 (38.81)	1321 (39.15)		124 (38.27)	131 (40.43)		
ASA, n (%)			<0.001			0.233	0.048
<3	262 (78.21)	2184 (64.73)		252 (77.78)	239 (73.77)		
≥3	73 (21.79)	1190 (35.27)		72 (22.22)	85 (26.23)		
BMI (kg/m2), median [IQR]	23.95 [21.22,26.88]	23.99 [20.90,27.06]	0.097	23.99 [21.22,26.91]	23.73 [20.58,26.35]	0.592	0.007
Tumor site, n (%)			<0.001			0.625	0.010
Left colon	216 (64.48)	1825 (54.09)		208 (64.20)	202 (62.35)		
Right colon	119 (35.52)	1549 (45.91)		116 (35.80)	122 (37.65)		
Tumor size, n (%)			0.003			0.931	0.003
<5 cm	124 (37.01)	1534 (45.47)		95 (29.32)	96 (29.63)		
≥5 cm	211 (62.99)	1840 (54.53)		229 (70.68)	228 (70.37)		
Differentiation grade, n (%)			0.642			0.517	0.027
Well/moderate	253 (75.52)	2509 (74.36)		244 (75.31)	251 (77.47)		
Poor/undifferentiated	82 (24.48)	865 (25.64)		80 (24.69)	73 (22.53)		
Histology type, n (%)			0.021			0.404	0.026
NMAC	299 (89.25)	3149 (93.33)		290 (89.51)	287 (88.58)		
MAC	21 (6.29)	129 (3.82)		19 (5.86)	15 (4.63)		
SRCC	15 (4.48)	96 (2.85)		15 (4.63)	22 (6.79)		
Clinical T category, n (%)			<0.001			0.207	0.019
cT3b	194 (53.91)	2712 (80.38)		193 (59.57)	184 (56.79)		
cT4a	61 (18.21)	492 (14.58)		61 (18.83)	79 (24.38)		
cT4b	80 (23.88)	170 (5.04)		70 (21.60)	61 (18.83)		
Clinical N category, n (%)			0.614			0.507	0.012
cN0	167 (49.85)	1711 (50.71)		162 (50.00)	149 (45.99)		
cN1	99 (29.55)	1041 (30.85)		97 (29.94)	110 (33.95)		
cN2	69 (20.60)	622 (18.44)		65 (20.06)	65 (20.06)		
Surgical approach, n (%)			<0.001			0.052	0.105
Open	271 (80.90)	2264 (67.10)		264 (81.48)	282 (87.04)		
Laparoscopic	64 (19.10)	1110 (32.90)		60 (18.52)	42 (12.96)		
Postoperative combination chemotherapy			<0.001			0.529	0.038
No	38 (11.34)	881 (26.11)		38 (11.73)	33 (10.19)		
Yes	297 (88.66)	2493 (73.89)		286 (88.27)	291 (89.81)		

Abbreviations: ASA, American society of Anesthesiologists physical status classification system; AD, adenocarcinoma; BMI, body mass index; IQR, inter quartile rang; MAC, mucinous carcinoma; PSM, propensity score-matching; SRCC, signet ring cell carcinoma.

In terms of patient demographics characteristics, patients in the NAC group were more likely to be of younger age (71.04% vs. 55.57%, *p* < .001) and had more rates of ASA <3 (78.21% vs. 64.73%, *p* < .001). In terms of tumor features, patients in the NAC group were more likely to have a cT4b tumor category (23.88% vs. 5.04%, *p* < .001) and left colon (64.48% vs. 54.09%, *p* < .001). In addition, patients in the NAC group had less NMAC (89.25% vs. 93.33%, *p* = .021) and larger tumor size (62.99% vs. 54.53%, *p* = .003) compared to those in the AC group. In terms of treatment, there were still more patients in the NAC group received open surgery (80.90 vs. 67.10, *p* < .001) and postoperative combination chemotherapy compared with the AC group (88.66 vs. 73.89, *p* < .001).

After propensity score matching for age, gender, ASA score, BMI, tumor site, tumor size, differentiation grade, histology, cT, cN, surgical approach and postoperative combination chemotherapy, 11 patients in the NAC group and 3050 patients in the AC group were excluded because no matching counterpart was found with a maximum caliber of 0.01. Ultimately, 324 match patient pairs were remained with no significant differences in the baseline characteristics between two groups, and SMD of the majority of variables included in PSM reduced to less than 0.1, demonstrating a good balance between two groups (Table 1).

### Survival outcomes in the matched cohort

**Figure 2.**
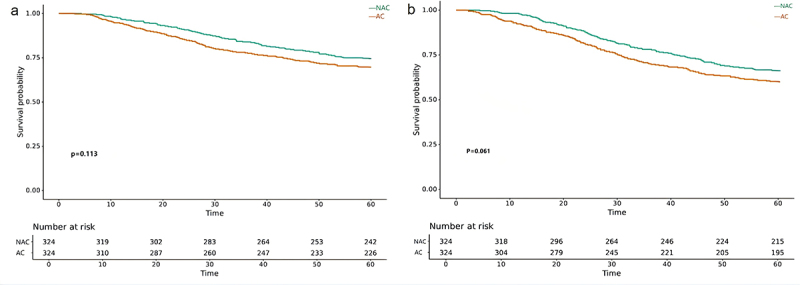
The OS and DFS in LACC patients with or without NAC. (a) Kaplan-Meier curve for OS stratified by NAC. (b) Kaplan-Meier curve for disease free survival stratified by NAC.

**Figure 3.**
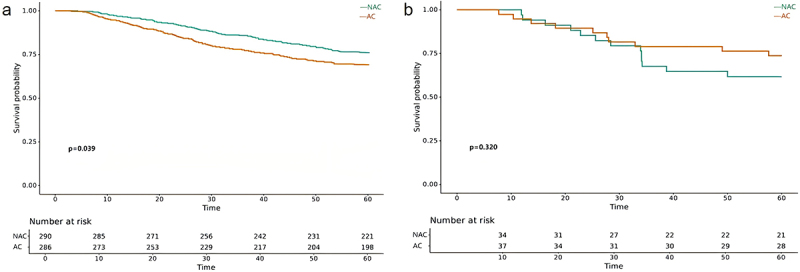
Kaplan-Meier analysis comparing OS (*p* value is for 5-year OS) of NAC group and AC group by histological types. (a) NMAC, (b) MAC/SRCC.

**Figure 4.**
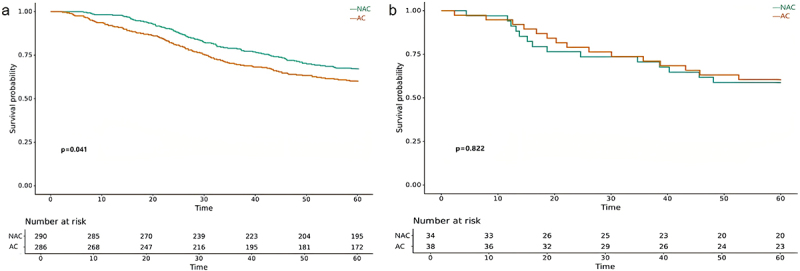
Kaplan-Meier analysis comparing DFS (*p* value is for 5-year DFS) of NAC group and AC group by histological types. (a) NMAC, (b) MAC/SRCC.

The median follow-up was 57 (4–105) months. The 5-year OS and DFS rates for the entire cohort were 72.2% and 63.3% respectively. Figure 2 depicted the K-M survival curves for NAC group and AC group after PSM. As shown, the OS (Figure 2a) and DFS (Figure 2b) were not significantly different between the two groups with a 5-year OS rate of 74.7% and 69.8% (*p* = .113) and 5-year DFS rate of 66.4% and 60.2% (*p* = .061). However, when considering the tumor histology type, patients with NMAC treated with NAC had an improved 5-year OS rate compared to patients treated with AC (76.3% vs. 69.2%, *p* = .039) (Figure 3a), and the 5-year DFS rate was also significantly different between the two treatment groups (67.2% vs. 60.1%, *p* = .041) (Figure 4a). Conversely, there was no significant difference in long-term survival outcomes between the two treatment groups among patients with MAC or SRCC (5-year OS rate: 61.8% vs. 73.7%, *p* = .320; 5-year DFS: 58.8% vs. 60.5%, *p* = .822) (Figure 3b,4b).

### Landmark analysis

**Figure 5.**
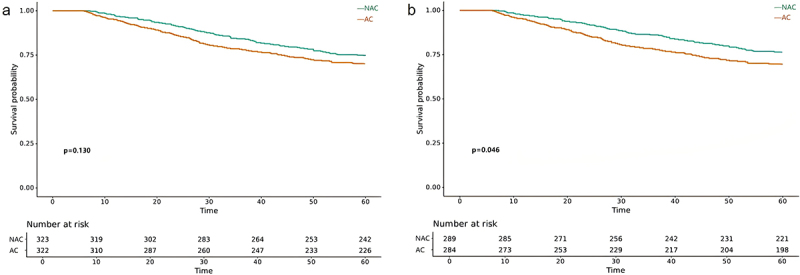
Kaplan-Meier analysis comparing OS (*p* value is for 5-year DFS) of NAC group and AC group by histological types in landmark cohort. (a) LACC, (b) NMAC.

Figure 5 depicted the K-M survival curves of OS for NAC group and AC group in landmark analysis. As shown, the OS were not significantly different between the two groups with a 5-year OS rate of 74.9% and 70.2% (*p* = .130) (Figure 5a). However, when considering the tumor histology type, patients with NMAC treated with NAC had an improved 5-year OS rate compared to patients treated with AC (76.5% vs. 69.7%, *p* = .046) (Figure 5b).

## Discussion

To our knowledge, this study is the first to evaluate the long-term effects of NAC in patients with LACC across different histological subtypes. The results suggest that, compared to patients who received AC alone, those with NMAC who underwent NAC showed significant improvements in both 5-year overall OS and DFS. However, for patients with MAC or SRCC, no significant differences in 5-year OS or DFS were observed between the two chemotherapy regimens.

NAC has clearly proven survival beneficial effects in multiple solid tumor types and has been incorporated into the multidisciplinary management of many advanced gastrointestinal cancers such as esophageal, gastric and rectal cancers.^6–8^ However, it has not been considered a common practice in the non-metastatic LACC yet. Previous studies have consistently confirmed the safety and feasibility of NAC in patients with LACC. Despite this, most published studies, including randomized controlled trials, have failed to demonstrate a significant impact of NAC on the long-term oncological outcomes in LACC.^18,22–24^ A major issue in this regard is the lack of consensus on the definition of subgroups of patients who are most likely to benefit from NAC.

Some of the few available studies regarding NAC found that CC with different molecular subgroups may not be equivalent in term of response to NAC. In a recent study, Dehal et al reported that there was a significant survival difference between patients undergoing NAC and those not undergoing NAC in T4b stage CC (HR 0.77, 95% CI 0.60–0.98; *p* = .04), rather than T3 or T4a.^18^ Similar results were also reported in the studies of McCahill and Smith et al.^25,26^ Their studies considered that NAC is only a promising potential choice for T4b CC, and supported by the National Comprehensive Cancer Network (NCCN).^27^ Furthermore, the Danish phase II randomized controlled trial and the British FOxTROT trial respectively found that the frequency of BRAF mutations and the status of microsatellite instability (MSI) may have also influenced the response of CC patients to chemotherapy and induced interpretation biases for survival analysis.^23,24^ Therefore, it may be beneficial to consider the different molecular subtypes of CC when deciding whether to undergo NAC.

Tumor histological type, as an important prognostic indicator, plays a crucial role in the formulation of personalized treatment plans for various cancers. However, in patients with CC, in addition to the well-established association of SRCC with poorer prognosis and resistance to therapy, histological type plays a limited role in treatment decision-making. Histopathological studies show that NMAC, characterized by glandular architecture, accounts for more than 90% of CC.^28^ The World Health Organization (WHO) classification also lists other histological variants of CC, such as mucinous, signet ring cells, spindle cells, and undifferentiated CC et al. Among them, MAC, as a rare and distinct subtype of colonic adenocarcinoma, accounts for 5–10% of all primary CC.^29,30^ The WHO definition of MAC is ‘‘an adenocarcinoma in which a substantial amount of mucin (more than 50% of the tumor) is retained within the tumor.^31^ This subtype differs from SRCC, which is characterized by abundant intracytoplasmic mucin that displaces the nucleus to the cell periphery, showing signet ring features in more than 50% of tumor cells.^32^ Many studies have found that the various histological CC subtypes exhibit different biologic behavior with variable outcomes. Studies have shown that compared with non-mucinous adenocarcinoma (NMAC), MAC is more common in younger females and is more likely to be associated with advanced stage at presentation, proximal colon high-degree microsatellite instability (MSI-H), and BRAF mutations.^33,34^ Moreover, MAC also had a higher rate of lymph node involvement and peritoneal spread.^35^ Many clinicopathological features of SRCC were also significant distinct with NMAC, such as an older age at onset, poorer grade of differentiation, advanced stage and more frequently infiltration into lymphatics and nodes.^32^ However, because most clinical trials on NAC do not yet differentiate treatment for LACC based on histological type, it remains unclear whether histology should influence the treatment decisions for NAC. Moving from this background, we conducted this largest population-based study to date to evaluate the long-term efficacy of NAC in LACC patients based on the histological type.

Indeed, the association between histological types and the efficacy of AC in LACC has been studied. In 2011, a retrospective study which involved 1,025 patients with stage II/III CC by Catalano et al found that SRCC was less responsive to chemotherapy in comparison to NMAC.^35^ Recently, the similar result was also validated by Jiang et al.^32^ Moreover, a group from Tongde Hospital of Zhejiang Province found that MAC was also less responsive to AC compared to NMAC in stage III CC.^34^ The results of multivariate Cox analyses showed that the receipt of chemotherapy was independently correlated with 46.0% decreased risk of colon cancer-specific mortality compared with non-chemotherapy group in NMAC, and this number had fallen to 37.7% in MAC. This finding was supported by several previous studies.^36,37^ In the present study, we observed that NMAC was more sensitive to NAC compared to MAC and SRCC in patients with LACC. The K-M survival analysis showed that the long-term prognosis of NMAC patients with the receipt of NAC was significantly better than those without the receipt of NAC. However, in LACC patients with MAC or SRCC histology, the OS and DFS rates of those receiving NAC were similar to those not receiving NAC, and the difference in survival was not statistically significant. The poor response to NAC in MAC and SRCC may be due to the relative hypoxic environment caused by reduced blood supply. Histopathological studies have shown that MAC and SRCC have lower microvessel density compared to NMAC, which may impair drug sensitivity due to decreased drug transport in the tumor microcirculation.^38^

It has been shown in several studies that different subtypes of MAC exist based on molecular alterations. Liu et al. distinguished MAC based on MSI status and reported that MSI-high MACs were usually low-stage tumors with a good prognosis and predictive of a sensitive response to chemotherapy.^39^ In addition, a recent study has linked mucus histology to a unique subgroup of CC, which displayed abnormal DNA hypermethylation, namely the CpG island methylation phenotype (CIMP).^40^ They found that 5-FU adjuvant chemotherapy did not provide clinical benefits in CIMP patients. Therefore, considering the above analysis, we conclude that to accurately investigate the prognostic value of NAC in patients with MAC, the impact of molecular alterations, such as MSI status and CIMP, should be further taken into account. However, the relatively small number of MAC/SRCC patients in this study prevented subgroup analysis, and further evaluation with a larger dataset is necessary.

The advantages of this study are as follows: Firstly, this study employed PSM to mitigate confounding effects, significantly enhancing the similarity of baseline characteristics between the neoadjuvant and adjuvant chemotherapy groups, thereby improving the comparability between the treatment and control groups. This approach reduced confounding bias in the survival analysis, thereby increasing the accuracy and reliability of the results. Second, the population-based design enhances the generalizability of the findings. Third, the primary outcome of the present study was OS. The choice of this outcome can avoid all possible misleading conclusions related to other outcomes. Finally, the sufficient follow-up period strengthens the robustness of our conclusions.

There were several limitations in our study. First, as a retrospective observational study, it is subject to inherent limitations that may lead to unavoidable biases or confounding factors. Second, a potential selection bias may be introduced by selecting only patients with an R0 resection. Therefore, future studies should include patients with varying resection statuses to more comprehensively evaluate the long-term effects of neoadjuvant chemotherapy. Third, although we employed a dichotomous age classification (≤65 years and >65 years) to simplify the analysis, this approach may result in the loss of some patient data or information. Specifically, reducing age to two categories may not fully capture the complex effects of age on treatment response. Fourth, using the time of diagnosis as the starting point for OS and DFS may introduce immortal time bias. However, the further landmark analysis effectively controlled this bias, thereby enhancing the reliability and accuracy of the results and yielding a more precise estimate of the treatment effect. Finally, the relatively small number of MAC/SACC patients after PSM may limit the generalizability of the statistical results.

## Conclusion

In conclusion, our study suggests that the prognostic value of NAC in patients with LACC varies according to histological subtype. Specifically, MAC and SRCC appear to be less responsive to NAC than NMAC, and NMAC may serve as a predictor of improved long-term survival with NAC in patients with LACC. Further large-scale prospective studies are needed to confirm whether histological subtype can predict the efficacy of NAC in patients with LACC.

## Data Availability

The datasets generated during and/or analyzed during the current study are available from the corresponding author on reasonable request.

## Abbreviations

AC: adjuvant chemotherapy
CC: colon cancer
cT: clinical T category
cN: clinical N category
DFS: disease-free survival
IQR: interquartile range
K-M: Kaplan-Meier
LACC: locally advanced colon cancer
MAC: mucinous adenocarcinoma carcinoma
NAC: neoadjuvant chemotherapy
NMAC: non-mucinous adenocarcinoma carcinoma
OS: overall survival
PSM: propensity score matching
SRCC: signet ring cell carcinoma
